# The characterization of collagen-based scaffolds modified with phenolic acids for tissue engineering application

**DOI:** 10.1038/s41598-023-37161-6

**Published:** 2023-06-20

**Authors:** Beata Kaczmarek-Szczepańska, Izabela Polkowska, Marcin Małek, Janusz Kluczyński, Katarzyna Paździor-Czapula, Marcin Wekwejt, Anna Michno, Anna Ronowska, Anna Pałubicka, Beata Nowicka, Iwona Otrocka-Domagała

**Affiliations:** 1 Department of Biomaterials and Cosmetics Chemistry, Faculty of Chemistry, Nicolaus Copernicus University in Torun, Gagarina 7, 87-100 Toruń, Poland; 2 Department and Clinic of Animal Surgery, Faculty of Veterinary Medicine, University of Life Sciences in Lublin, Akademicka 13, 20-950 Lublin, Poland; 3 Faculty of Civil Engineering and Geodesy, Military University of Technology, ul. Gen. Sylwestra Kaliskiego 2, 00-908 Warsaw, Poland; 4 Faculty of Mechanical Engineering, Military University of Technology, ul. Gen. Sylwestra Kaliskiego 2, 00-908 Warsaw, Poland; 5 Department of Pathological Anatomy, Faculty of Veterinary Medicine, University of Warmia and Mazury, Oczapowskiego 13, 10-719 Olsztyn, Poland; 6 Department of Biomaterials Technology, Faculty of Mechanical Engineering and Ship Technology, Gdańsk University of Technology, Gabriela Narutowicza 11/12, 80-229 Gdańsk, Poland; 7 Department of Laboratory Medicine, Medical University of Gdańsk, Marii Skłodowskiej-Curie 3a, 80-210 Gdańsk, Poland; 8 Department of Laboratory Diagnostics and Microbiology With Blood Bank, Specialist Hospital in Kościerzyna, Alojzego Piechowskiego 36, 83-400 Kościerzyna, Poland

**Keywords:** Health care, Materials science

## Abstract

The aim of the experiment was to study the morphology of collagen-based scaffolds modified by caffeic acid, ferulic acid, and gallic acid, their swelling, and degradation rate, as well as the biological properties of scaffolds, such as antioxidant activity, hemo- and cytocompatibility, histological observation, and antibacterial properties. Scaffolds based on collagen with phenolic acid showed higher swelling rate and enzymatic stability compared to scaffolds based on pure collagen, and the radical scavenging activity was in the range 85–91%. All scaffolds were non-hemolytic and compatible with surrounding tissues. Collagen modified by ferulic acid showed potentially negative effects on hFOB cells as a significantly increased LDH release was found, but all of the studied materials had antimicrobial activity against *Staphylococcus aureus* and *Escherichia coli*. It may be assumed that phenolic acids, such as caffeic, ferulic, and gallic acid, are modifiers and provide novel biological properties of collagen-based scaffolds. This paper provides the summarization and comparison of the biological properties of scaffolds based on collagen modified with three different phenolic acids.

## Introduction

Over the course of several decades, along with changing trends, biomaterials have been a particular subject of research. Biomaterials have direct contact with living tissue or fluids over a period of time or permanently, leading to an improvement in conditions of or saving a person's life. Biomaterials may be metallic, ceramic, carbon-based, or polymeric. Each of them has different properties and is intended for another purpose. Depending on the biomaterial itself and the patient's individual characteristics, the response of the body may vary. In case of little or no interaction with the surrounding tissue materials are called as bioinerts, while those that are characterized by stimulation and good tissue bonding are known as bioactive materials. The intended use of the material is another important point that should be considered in the existence of the aforementioned factors. It is possible to indicate a number of materials that have functions in the various fields such as filling, anastomosis, and replacement of tissues or organs^1–3^. The main conditions are biocompatibility with the body, adequate mechanical properties, high wear resistance, the possibility of surface modification, integrity with the environment, blood compatibility, corrosion-resistance, sterilizability, and biodegradability. Advanced scientific research has led to the dynamic development of biomaterials and the creation of many structures applicable in a wide range of fields, such as dentistry, maxillofacial surgery, ophthalmology, and regenerative medicine, orthopedics, bioengineering, cardiology, and plastic surgery^4–6^.

Collagen is a protein widely used to fabricate biocompatible materials^7^. Collagen can be isolated from natural sources such as fungi, fish skin, and scales, as well as from rat tails, pig and beef tissues, and also from sea sponges, jellyfish, and egg capsules of the dogfish^8^. Collagen is one of the extracellular matrix proteins and as a biomaterial is used in a variety of connective tissue applications. This is mainly due to its exceptional properties such as excellent biocompatibility, high degradability into fully tolerable compounds, low inflammatory response, hemostatic properties, osteoconductivity as well as cell-binding ability^9–11^. Collagen-based materials are interesting to be studied as collagen has excellent biocompatibility. Further, it was verified by researchers that collagen scaffolds do not induce any cytotoxic effects and show higher cell viability than other similar materials (for example gelatin, hyaluronic acid, or glucan)^9^. Moreover, such collagen-based scaffolds are characterized by the ability to support the osteogenic differentiation of mesenchymal stem cells^12^. The main disadvantage of collagen is its low stability after implantation. Thereby, it cannot be used in regenerative medicine without previous modifications^13,14^.

One of the methods to improve the properties of collagen-based scaffolds is the use of phenolic compounds as cross-linkers or modifiers^15^. Phenolic compounds are widespread in nature^16^. They are found mainly in fruits and vegetables which are byproducts of plant metabolism. Especially rich in these compounds are chokeberry, dark grapes, blueberries, strawberries, raspberries, blackcurrants, apples, oranges, and vegetables, i.e. lettuce, broccoli, legumes, onions, peppers, tomatoes, and more. They are characterized by a diversified structure, molecular weight as well as biological, chemical, and physical properties. Their natural character is an overwhelming advantage that attracts scientists’ interest in their use as modifiers for biopolymers^17,18^. However, their high concentration may be harmful and the biocompatibility of collagen materials modified by phenolic acids has to be considered.

The study aimed to summarize and compare the biocompatibility of scaffolds based on collagen modified with phenolic acids such as caffeic acid, ferulic acid, and gallic acid, as well as to assess their antibacterial potential. Phenolic acids were selected from the literature as the most frequently proposed protein modifiers. The biocompatibility was evaluated as the response of human blood, an in vitro study with human osteoblasts cells, and in in vivo study by the implementation of biomaterials in animals. Thereby, the safety of the application of the mentioned scaffolds has been discussed. In the literature, there is a lack of in vivo studies of the proposed scaffolds dedicated to tissue engineering. Thereby, this paper includes the study of the implantation of scaffolds into the animal model.

## Results

### Physicochemical properties

#### Scanning electron microscope

**Figure 1:**
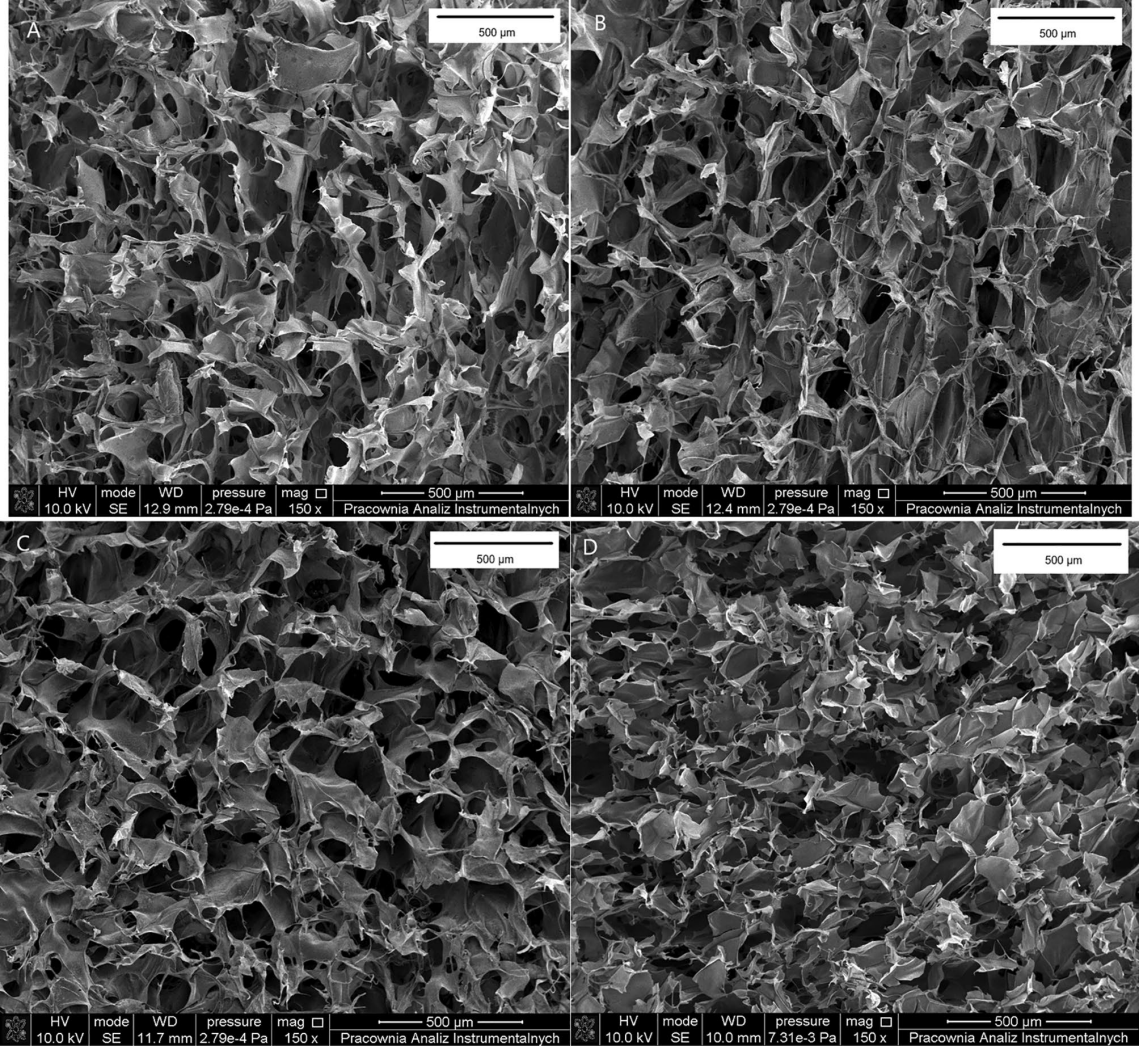
The SEM images of scaffolds based on (**A**) collagen, (**B**) collagen with caffeic acid, (**C**) collagen with ferulic acid. (**D**) Collagen with gallic acid.

All the scaffolds showed porous morphology with open interconnected pores (Fig. 1) with pore diameter of 112 (± 14.2) µm for collagen, 108 (± 21.1) µm for collagen modified by caffeic acid, 162 (± 17.8) µm for collagen modified with ferulic acid, and 115 (± 25.6) µm for collagen modified with gallic acid.

#### Swelling rate

**Figure 2:**
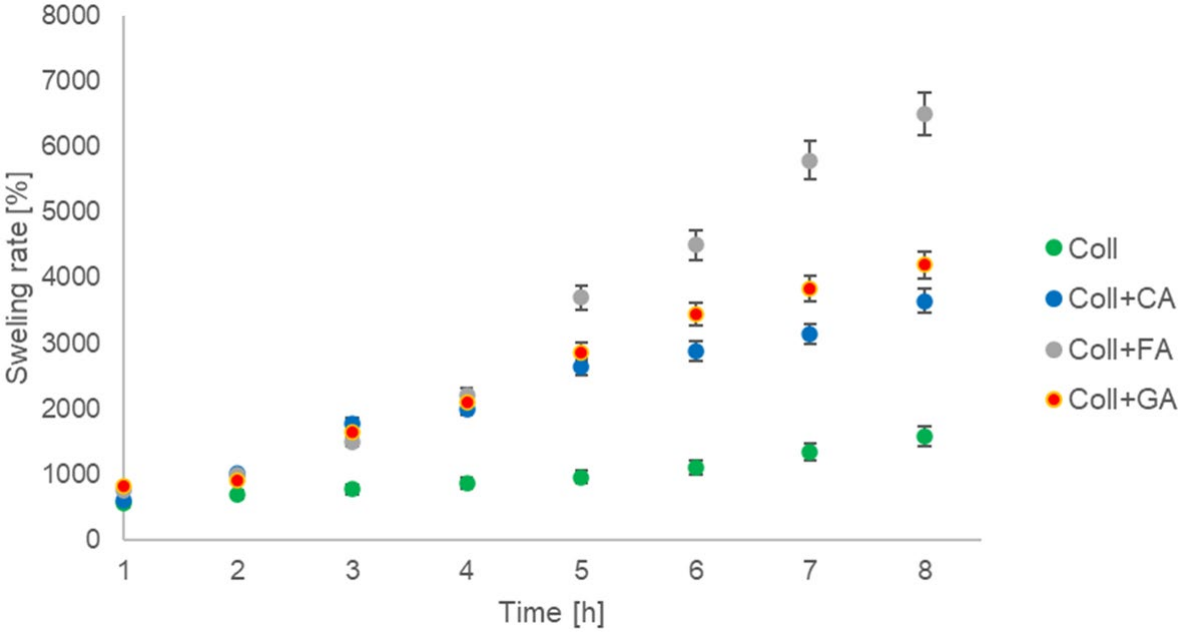
The swelling rate for scaffolds based on collagen (Coll), collagen with caffeic acid (Coll + CA), ferulic acid (Coll + FA), and gallic acid (Coll + GA) (n = 3, data are expressed as the mean ± SD).

The swelling rate of the scaffolds based on collagen and collagen modified by the addition of caffeic acid, ferulic acid, and gallic acid is shown in Fig. 2. The highest swelling rate in time was shown by collagen scaffolds modified with ferulic acid. The lowest swelling rate was noticed for the scaffolds based on collagen without modification.

#### Biodegradation

**Figure 3:**
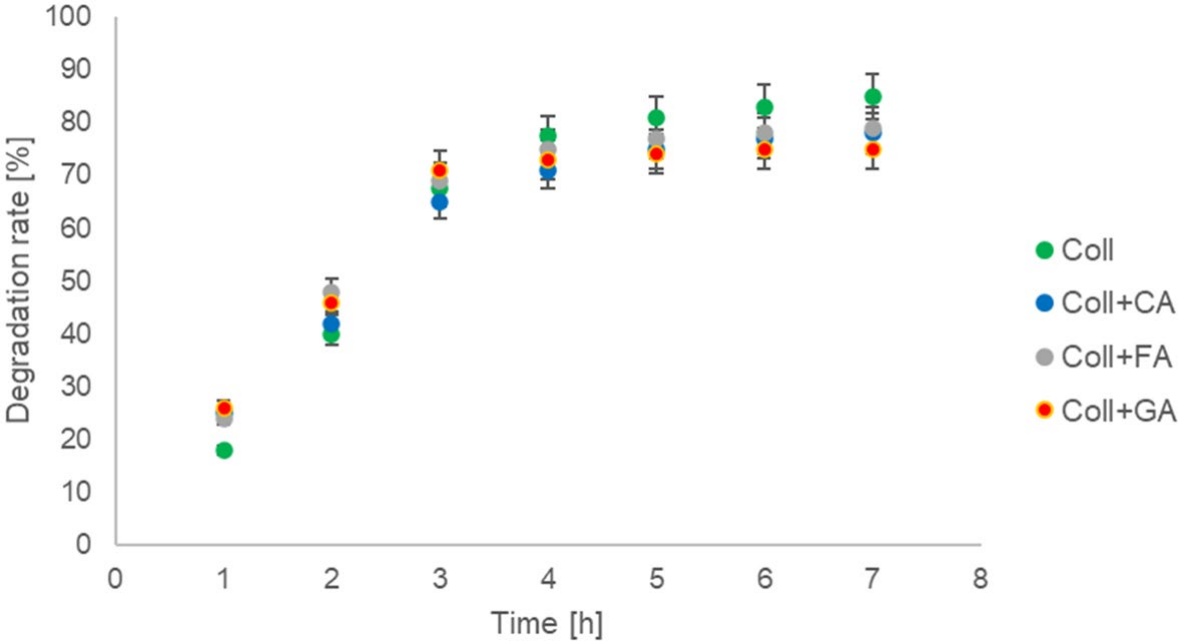
The biodegradation rate for scaffolds based on collagen (Coll), collagen with caffeic acid (Coll + CA), ferulic acid (Coll + FA), and gallic acid (Coll + GA) (n = 3, data are expressed as the mean ± SD).

The biodegradation study was carried out, and the results are shown in Fig. 3. Collagen-based scaffolds without phenolic acids showed the highest degradation rate. The addition of caffeic acid, ferulic acid, or gallic acid decreases the degradation rate. Thereby, it can be assumed that the addition of phenolic acids improves the stability of collagen-based scaffolds.

#### Antioxidant properties

Biomaterials with antioxidant activity are valuable in regenerative medicine as they are able to protect cells and tissues against harmful oxidation processes^19^. Collagen-based scaffolds without modification do not have antioxidant activity (negative value). Phenolic acids addition results in activity in the range of 85–91% (Table 1). The highest activity was noticed for scaffolds with ferulic acid addition (Coll + FA).

**Table 1:** The radical scavenging activity obtained for collagen scaffold modified with phenolic acids (n = 4, average ± SD, *significantly different from Coll+CA, # from Coll+FA (p < 0.05)).

Sample	RSA [%]
Coll	− 0.57 ± 0.09
Coll+CA	90.23 ± 0.15^#^
Coll+FA	91.58 ± 0.11*
Coll+GA	85.29 ± 0.22*^#^

### In vitro validation

#### Hemo- and cytocompatibility

**Figure 4:**
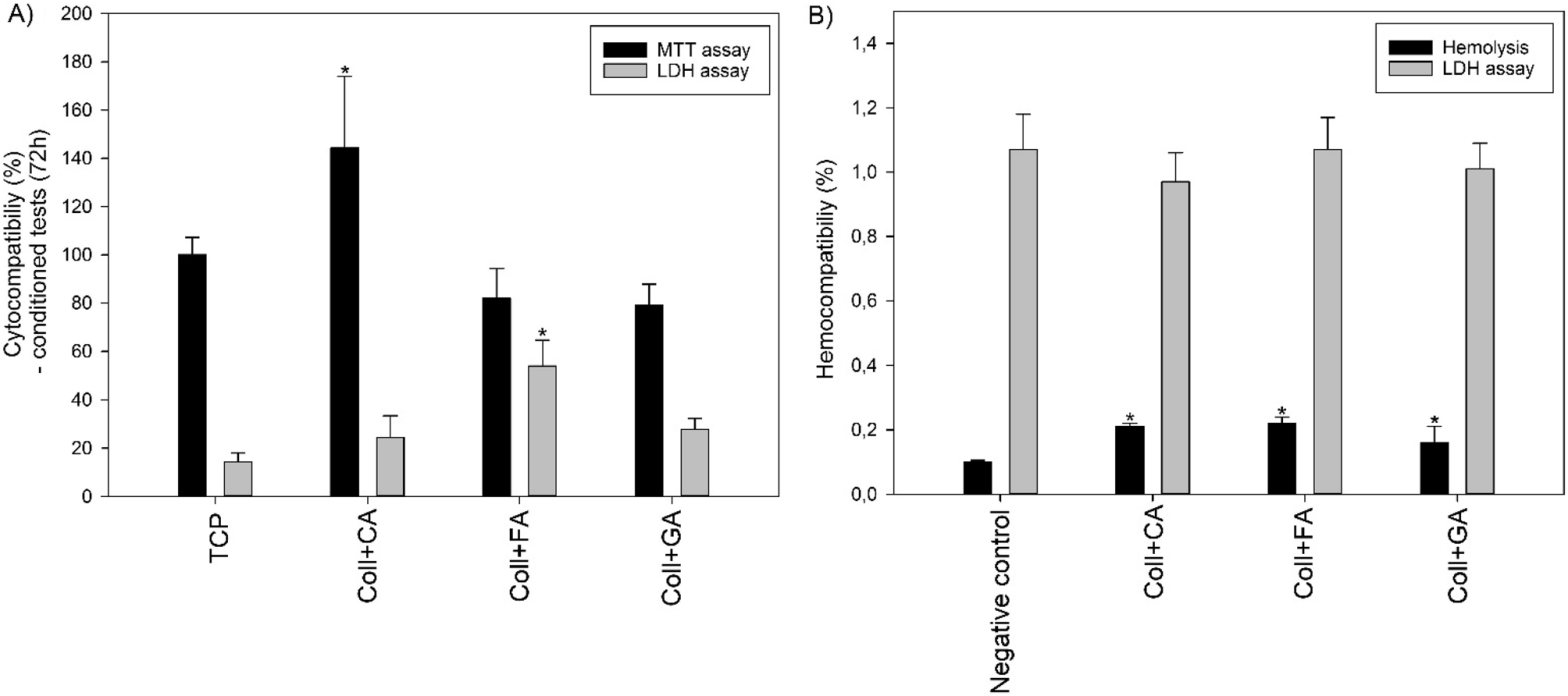
The effect of developed scaffolds based on collagen modified with caffeic acid (CA), ferulic acid (FA), and gallic acid (GA) on cytocompatibility of hFOB 1.19 cells (expressed by MTT proliferation and lactate dehydrogenase release) after 72 h of culture and hemocompatibility of human erythrocytes (expressed hemolysis rate and lactate dehydrogenase release) after 24 h exposure to materials (n = 4, data are expressed as the mean ± SD,*significantly different from the respective controls (p < 0.05)).

All the modified collagen-based scaffolds may be considered biocompatible as no significant negative effect on the human erythrocytes and osteoblastic cells was observed (Fig. 4). The hemocompatibility studies (Fig. 4B) demonstrated a low degree of hemolysis (below 0.5%; relative to 100% positive control) and the release of LDH comparable to the control conditions. While cytocompatibility studies showed that neither, Coll+CA nor Coll+GA exerted cytotoxic effect on human osteoblastic cell line hFOB 1.19, because the LDH release was not elevated (Fig. 4A). On the other hand there was significant, ~ 44 (± 29.6)% increase of proliferation in the cells grown on CA enriched scaffolds. GA caused ~ 20 (± 8.85)% inhibition of cellular growth, however this change was not significant. Only scaffolds modified with FA were cytotoxic to the cells. There was significant ~ 35 (± 10.6)% increase of LDH release from damaged cells accompanied with ~ 20 (± 12.3)% fall of cell growth (Fig. 4A).

**Figure 5:**
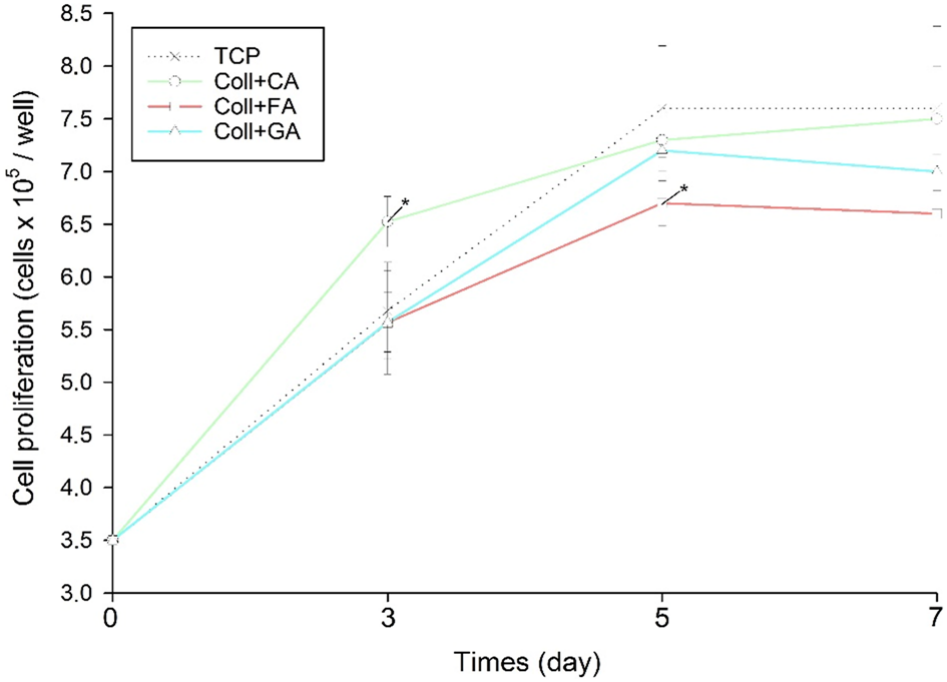
The effect of the developed scaffolds based on collagen modified with caffeic acid (CA), ferulic acid (FA), and gallic acid (GA) hFOB 1.19 cell proliferation (n = 4, data are expressed as the mean ± SD, *significantly different from Tissue Culture Plate (TCP) (p < 0.05)).

Human osteoblast cell proliferation was also monitored for seven days (Fig. 5). It was found that only scaffold Coll + FA inhibited cellular growth after a longer culture period (5 days). A significant positive effect on cell proliferation was also observed in the shorter culture time (3 days) for Coll + CA. Both of these results are consistent with the cytocompatibility study (Fig. 1) in which cell viability after 72 h was evaluated. Furthermore, there is a tendency for a slight slowdown in the proliferation grown in the presence of medium conditioned with scaffolds modified with FA and GA.

#### Antibacterial properties

**Figure 6:**
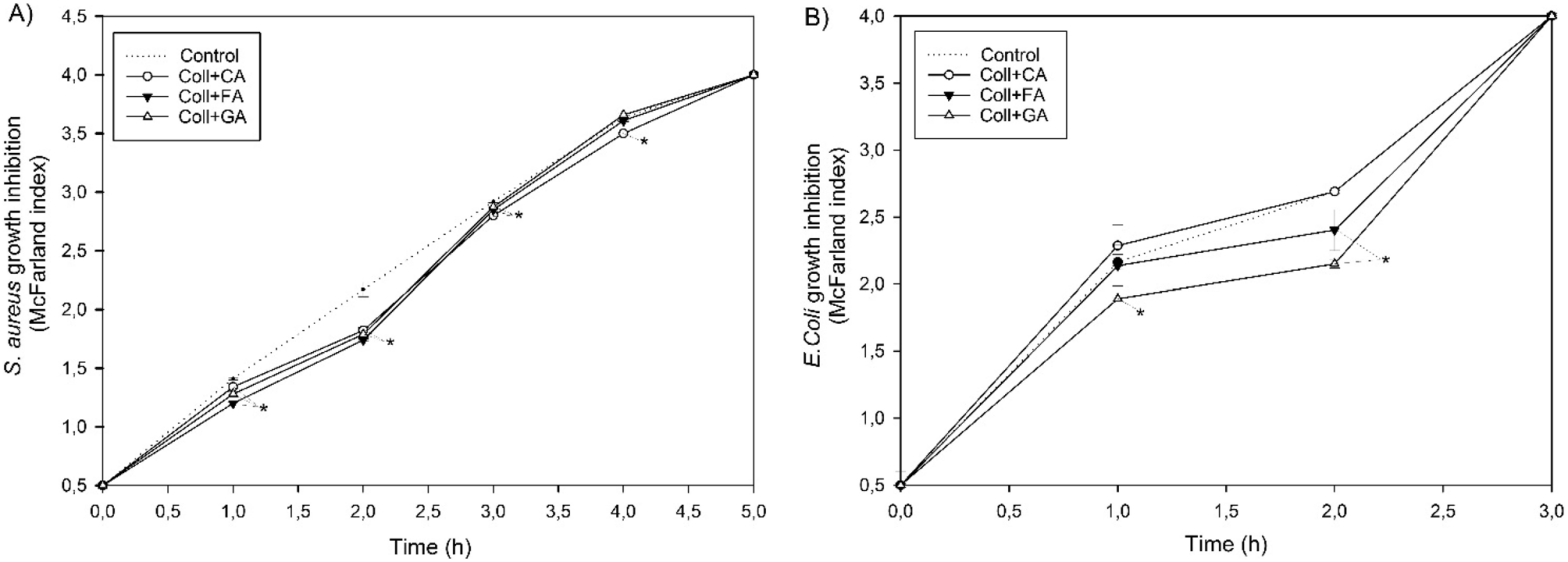
The effect of the developed scaffolds based on collagen with caffeic acid (CA), ferulic acid (FA), and gallic acid (GA) on bacterial growth inhibition determined by McFarland standard values specifying the number of selected bacteria during the incubation (n = 3, data are expressed as the mean ± SD, *significantly different from the control (p < 0.05)).

The scaffolds modified with phenolic acids slowed down the multiplication of bacteria in the bacterial medium, hence they were characterized by antibacterial properties (Fig. 6). In the case of *Staphylococcus aureus* (A), all the materials significantly inhibited its multiplication in the first three hours of incubation, which may be related to the release of decomposition products such as released phenolic acid from the matrix, with particular effectiveness for Coll + CA (bacteria multiplication reduced by 15%, 5.5% and 3.8% on 2, 3, 4 h, respectively). However, in the case of *Escherichia Coli* (B), only scaffolds with FA and GA showed antibacterial properties, and gallic acid had the greatest effect on slowing down the multiplication of bacteria (14.5% and 25% on 1 and 2 h, respectively).

### In vivo validation and histological analysis

**Figure 7:**
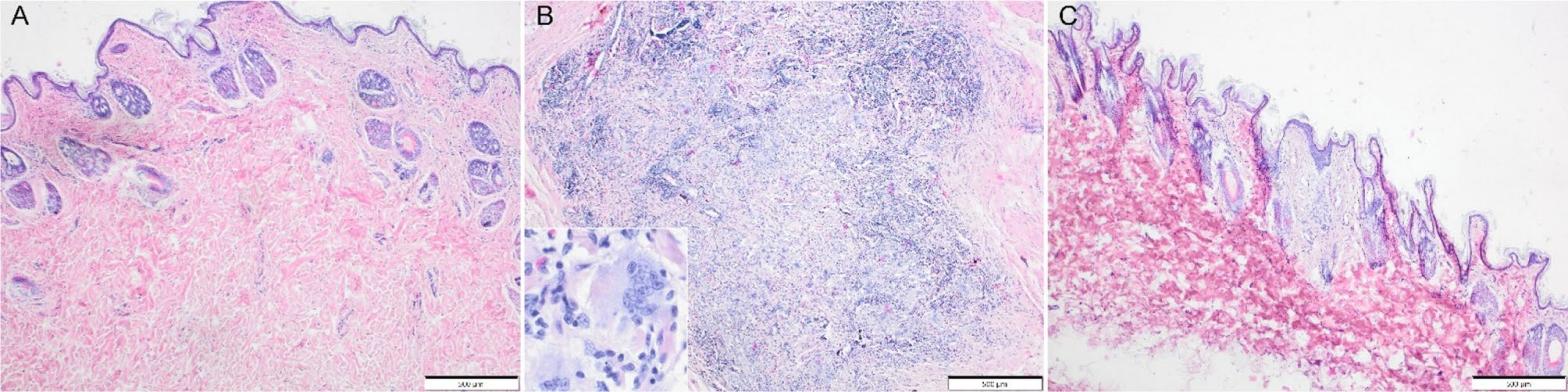
Tissues collected after implantation of collagen-based scaffolds in New Zealand rabbits (HE). (**A**) Collagen with caffeic acid (Coll + CA). There is mild fibrosis, visible in the cutis propria. Adnexa is reduced in number in this area. (**B**) Collagen with ferulic acid (Coll + FA). There is a large focus on macrophages, multinucleated giant cells, fagocytosing pink material (scaffold elements—inset), and numerous lymphocytes. (**C**) Collagen with gallic acid (Coll + GA). There is a focal proliferation of vascularized connective tissue just beneath the epidermis, moderately acanthotic.

In samples collected from the site of scaffold Coll+CA implantation, mild focal cutaneous fibrosis and focal regeneration of the cutaneous muscle were observed, but indiscernible grossly. No implant fragments nor inflammatory reaction was observed (Fig. 7A). In samples collected from the site of the Coll+FA scaffold implantation, foci of macrophages, quite numerous multinucleated giant cells (of foreign body type), variably numerous lymphocytes, and single heterophils infiltration were observed within the subcutaneous tissue, with a concurrent proliferation of the connective tissue (Fig. 7B). These findings, visible grossly as focal thickening of the skin, indicate the advanced implant resorption. In samples collected from the site of Coll+GA scaffold implantation, a focal proliferation of well-vascularized, collagen-rich connective tissue with hyalinization within the skin was evident. The epidermis above was moderately acanthotic (Fig. 7C). These findings were indiscernible grossly. No implant fragments nor inflammatory reaction was observed.

## Discussion

Phenolic acids such as caffeic, ferulic, and gallic acid were added to the collagen solution during the collagen-based materials fabrication process. Highly porous materials (scaffolds) were obtained by the freeze-drying method because of solvent evaporation. They were homogenous and flexible, without any damage. The expected properties of the obtained materials were their antioxidant activity and biocompatibility.

The swelling and degradation rate are important to be considered for the application of biomaterials in tissue engineering. The scaffolds’ swelling ability may be associated with increased diffusion of signaling molecules and nutrients into the material. Scaffolds with high swelling rate might facilitate the transportation of nutrients and metabolites^20^. Moreover, the high swelling ability of scaffolds can reduce the risk of skin damage^21^. Our studies showed the increase of swelling rate associated with the presence of phenolic acid in the collagen-based scaffolds.

Another important factor is the degradation rate of a scaffold. The scaffold should degrade at a speed that will allow for the subsequent native tissue regeneration^22^. The studies of the degradation rate in the presence of collagenase showed that the addition of phenolic acids decreases the degradation rate. It suggests that they improve the stability of scaffolds against enzymatic degradation. Similar results for collagen-gallic acid complexes were obtained by Zhao et al.^23^. It was found that ferulic acid inhibits collagen self-association. Moreover, the enzymatic studies suggested that ferulic acid protects collagen from degradation^24^. Thereby, it can be confirmed that our results refer to the conclusions drawn by other scientists about the influence of phenolic acid on the enzymatic stability of collagen-based scaffolds.

Antioxidants are compounds that are able to eliminate harmful ROS excess and inhibit or even delay the oxidation processes. The implantation of biomaterials with antioxidant properties will help to avoid damage during the tissue regeneration process^25^. ROS may damage the extracellular matrix proteins due to their high chemical activity. Thereby, the antioxidant biomaterials may promote enhanced healing^26^. It was reported that phenolic acids are powerful antioxidants^27^. However, their use as polymeric modifiers may inhibit their effectiveness. Therefore, it is important to study the antioxidant properties of the fabricated materials. Bergonzi et al.^28^ reported phenolic compounds such as Curcumin as effective antioxidants. Its addition allowed to control wound oxidative stress. The DPPH method was determined as the RSA of ALG/HELP films with dispersed curcumin and ranged from 66.2 ± 4.7% to 84.9 ± 4.5%. Chitosan/collagen hydrogels were modified by caffeic acid by Thongchai et al.^29^. The addition of caffeic acid resulted in the RSA of 28% for 5% addition, 62% for 10%, 84% for 20% and 30%. The studies of Coll+CA, Coll+FA, and Coll+GA provide the RSA of over 85%. It suggests that studied phenolic acids, such as caffeic acid, ferulic acid, and gallic acid, are powerful antioxidants, that remain active even after mixing with collagen.

Collagen 2D or 3D structures (such as films, gels or scaffolds) may be obtained by different methods, however cross-linking is often used for the improvement of their biostability, mechanical properties, and water resistance^30^. Currently, there is extensive research on various modifying agents and also phenolic acids, used in this study, may be used as effective modifiers for collagen. In our previous study, we found that different modifying agents may contribute to better cytocompatibility of collagen-based materials, for example, tannic acid and starch dialdehyde^31^. Hence, we assume that the addition of different phenolic acids, such as GA, FA, and CA would have a different effect on the properties of the obtained scaffolds, especially in vitro and in vivo response^8^. Literature reports confirm the biocompatibility of the used components. For example, Bam et al. showed that scaffolds cross-linked by dialdehyde chitosan with gallic acid have good cytocompatibility^32^. Chahardoli et al. reported that caffeic acid-based TiO_2_ nanoparticles have no hemolytic potential and Krishnamoorthi et al. found that caffeic acid/chitosan polymer exhibit good cytocompatibility^33,34^. Choi et al. obtained results showing that ferulic acid at a low concentration (300 µL/mL) does not have to induce cytotoxicity^35^. Hence, we assume that all components used for our developed scaffolds should not adversely affect the cellular response.

However, here we found that various phenolic acids do not cause hemolysis, while they have a different effect on the viability and proliferation of osteoblastic cells. Caffeic acid significantly improved hFOB cells proliferation in shorter culture time, whereas gallic acid slightly slowed inhibited proliferation down, but without significant increase of cell death disturbance of cell viability. In turn, ferulic acid slightly slowed down the proliferation, especially significant after 5 days, and also increased cell mortality. It is worth emphasizing that all the phenolic acids in this study were used in equal content and with the same methodology, hence a different cellular response may be attributed to a given acid. Some research showed that caffeic acid may act as a therapeutic agent with antioxidant and anti-inflammatory properties^36^, which may explain its positive influence (after 3 days) on the cytocompatibility of collagen-based scaffolds. Our result is also in line with results shown in Ekueku et al.’s review regarding the positive effect of CA supplementation on bone remodeling^37^. For the other two phenolic acids, concentration may be an issue and as the scaffold degrades, acid compounds are released into the culture medium. Cho et al. found that with increasing release of GA concentration (50–500 μg/mL), the initially positive effect on cell proliferation decreased, but still without significant negative effect on cell viability—which we also observed here^38^. Moreover, He et al. and Prasad et al. showed that selected phenolic compounds, especially gallic acid and caffeic acid, may be used to inhibit human cancer cells proliferation^39^. Further, Ouimet et al. reported that ferulic acid-based polymers with glycol were characterized by high cytocompatibility only in relatively low concentrations released into culture media (up to 10 μg/mL). When the concentration at 100 μg/mL was tested, they observed a decrease in cell viability—which we observed here^40^. Therefore, it may be assumed that the released concentration of both GA and FA from dissolved scaffolds may exceed the safe dose for cells, especially for osteoblasts tested here, caused slowing down of their multiplication and lowering their survival rate.

The in vivo results are relatively consistent with in vitro study*.* Scaffolds based on Coll with CA and GA do not generate an inflammatory process, while for those with FA such a process was observed. Based on the histological assessment it may be assumed that collagen scaffolds modified by caffeic and gallic acid resorb faster and easier than scaffolds with ferulic acid. In such short time after the implantation, the tissue regeneration was not noticed and fibrosis occurred, but the adnexal structures were not restored. Based on the results obtained by in vivo study, investigating collagen scaffolds modified by caffeic or gallic acid would be recommended.

According to the literature, phenolic acids may have antibacterial properties. It depends on the lipophilicity and the electronic and charge properties of the polyphenols^41^. The modified scaffolds obtained here also showed a positive effect of inhibiting the multiplication of bacteria. During the degradation, immediately after the implantation of scaffolds, phenolic acids are released into the surrounding solution and with antibacterial ability may prevent the development of infection. We found that the addition of all phenolic acids was effective for *Staphylococcus aureus*, however, Coll+CA did not inhibit *Escherichia coli* in general. The obtained results are consistent with the studied carried out by Andtade et al.^42^. The conclusions made by researchers were: phenolics with longer alkyl side chains are more effective against Gram-positive bacterium and caffeic acid effectivity against bacteria depends on the length of the alkyl chain. Further, Borges et al. confirmed the antibacterial effectiveness of the gallic and ferulic acids and also found that they have various minimum inhibitory concentrations (MIC) and minimum bactericidal concentrations (MIB) depending on different bacteria (*P. aeruginosa*, *E. coli*, *S. aureus,* and *L. monocytogenes)*^43^. Our studies confirmed that phenolic acids added to the collagen matrix are a green and sustainable source of new broad-spectrum antimicrobial products. Furthermore, focusing on this application of scaffolds, it may be assumed that different phenolic acid should be used depending on the type of bacterial infection (Gram+ or Gram- bacteria). All tested scaffolds would be suitable for *S. aureus* classified as Gram+ bacteria, while Coll+CA does not have antibacterial properties against *E. Coli*, i.e. Gram- bacteria. These differences may be related to the cellular structure of the above bacteria, which determines the different permeability. Gram- bacteria have a thin peptidoglycan layer with a relatively simple cross-linking structure and they also possess an outer lipid membrane which makes them more resistant to external factors. However, in the case of Gram+ bacteria the layer is very thick and extensively cross-linked as well as additionally containing teichoic and lipoteichoic acids^44^. Further, there are also significant differences in the aspect of biofilm formation (its initiation, maturation and dispersal) by the above types of bacteria^45^.

## Methods

### Sample preparation

Ferulic acid (FA) and caffeic acid (CA) were purchased from ROTH company. Gallic acid (GA) was purchased from PolAura Company. Collagen (Coll) was isolated from the rat tail tendons. Collagen was freeze-dried and then dissolved at 1% concentration (in 0.1 M of acetic acid). The collagen solution was mixed with phenolic acid solutions such as ferulic acid, gallic acid, and caffeic acid. Each of the phenolic acids was dissolved separately in 0.1 M of acetic acid at 1% concentration. The phenolic acid solution was added to the collagen solution in the 10 w/w% ratios. The mixtures were mixed on the magnetic stirrer for 1 h at room temperature. Then they were frozen by using the lyophilizer (ALPHA 1–2 LDplus, CHRIST, − 20 °C, 100 Pa, 48 h). The 3D porous scaffolds were obtained and used for the biological assessment.

### Physicochemical properties

#### Scanning electron microscope

The morphology of the samples was studied using a scanning electron microscope (SEM; LEO Electron Microscopy Ltd, England). The samples were covered with gold before the analysis. Scanning electron microscope images were made with magnification of 150×.

#### Swelling rate

Scaffolds (dry with known weight, W_dry_) were immersed in PBS for 8 h to study the swelling behavior. After 1, 2, 3, 4, 5, 6, 7, and 8 h scaffolds were gently dried with the tissue paper and then weighted (W_wet_). The swelling rate was then calculated from the equation:

$$Swelling\,rate=\frac{({W}_{wet}-{W}_{dry})}{{W}_{dry}}*100\%$$

#### Biodegradation

For the degradation study, the scaffolds (with known initial weight, W_0_) were immersed in PBS containing collagenase (1 U/mL) and incubated at 37 °C for 7 days. The scaffolds were then washed with water, frozen, freeze-dried and weighted (W) after each day. The degradation rate was calculated from the equation:

$$Degradation\,rate=\frac{({W}_{0}-W)}{{W}_{0}}*100\%$$

#### Antioxidant properties

The DPPH reagent (2,2-Diphenyl-1-picrylhydrazyl, free radical, 95%; Alfa Aesar, Germany) was used to determine the antioxidant properties (RSA% = radical scavenging activity). The samples were placed in a 24-well plate and filled with 2 mL of a DPPH solution (250 µM solution in methyl alcohol), After 1 h, a spectrophotometric measurement was made at 517 nm (UV-1800, Shimadzu, Switzerland). The radical scavenging activity was calculated from the formula:

$$RSA\%=\frac{{Abs}_{DPPH}-{Abs}_{PB}}{{Abs}_{DPPH}}*100\%$$

where Abs_DPPH_ is the absorbance of the DPPH solution without contact with the material being tested; Abs_PB_ is the absorbance of the DPPH solution after contact with the material being tested.

### In vitro validation

#### Hemo- and cytocompatibility

The experiments on hemo- and cyto-compatibility of the scaffolds were conducted on human red blood cells (RBCs) and human osteoblasts cell lines (hFOB 1.19, ATCC RRID: CVCL 3708). The number of cells was estimated with a hemocytometer Superior CE (Marienfeld, Lauda-Königshofen, Germany). For the hemocompatibility study scaffolds in a cylinder form with the size of about 3 mm in diameter and 3 (± 0.02) mm thick were used, while for cytocompatibility scaffolds in cylinder form with a size of 13 mm in diameter and 3 (± 0.02) mm thick were used. Before testing, all specimens were sterilized by UV-light exposure for 30 min. The reagents, unless otherwise noted, were purchased from Merck kGaA (Germany).

RBCs were obtained from buffy coats highly contaminated with RBCs obtained as by-products of whole blood fractionations from the Regional Blood Centre in Gdańsk (Regional Blood Blank institutional permission M-073/17/JJ/11). Whole blood was collected from healthy volunteers in accordance with the Declaration of Helsinki under an approved Regional Bank review board protocol in standard acid citrate dextrose solutions. RBCs were fractionated according to the standards of Blood Banks (International Standard for the Blood Banks and Blood Transfusion Services; NACO: New Delhi, India, 2007). RBCs (3 × 10^9^ cells/mL) were placed in 2 mL tubes containing scaffolds (n = 4) and incubated at 37 °C for up to 24 h. The remaining blood samples were centrifuged at 100×*g* at room temperature for 3 min to let the erythrocytes sediment and the supernatants were taken for further research. The hemolysis was assessed spectrophotometrically at a wavelength of 540 nm with an Ultrospect 3000pro spectrophotometer (Amersham-Pharmacia-Biotech, Cambridge, UK). RBCs treated with 2% Triton were used as a positive control (i.e., 100% hemolysis) and RBCs incubated without films as a negative control. Results are presented as percentage of hemolysis according to the formula^46^:

$$Hemolysis\%=\frac{{Abs}_{sample}-{Abs}_{negative control}}{{Abs}_{positive control}-{Abs}_{negative control}}*100\%$$

where Abs_sample_ is the absorbance of the supernatant of sample incubated with the tested materials; Abs_negative control_ is the absorbance of the supernatant of sample incubated without the tested materials; Abs_positive control_ is the absorbance of the supernatant of sample incubated without the tested materials and treated with 2% Triton.

The hFOB cells were grown in a 1:1 mixture of Ham’s F12 Medium and Dulbecco’s Modified Eagle’s Medium (without phenol red), containing 1 mmol/L l-glutamine, 0.3 mg/mL genetin (G418) and 10% fetal bovine serum at 37 °C in an atmosphere containing 5% CO_2_. The extracts of the scaffolds were used to study their cytocompatibility and proliferation effect. Each scaffold (n = 4) was immersed in 2 mL of culture medium and conditioned for 24 h. Then, for cytocompatibility study, the cells were seeded at a density of 12 × 10^3^ cells on a 96-well plate and cultured for 24 h at standard conditions. After that time the medium was discharged and the conditioned medium was added for the next 72 h. The cell viability was evaluated after 72 h of culture using, the MTT assay (3-(4,5-dimethylthiazol-2-yl)-2,5-diphenyltetrazolium bromide). The medium was changed for the one supplemented with 0.06 mmol/L of MTT and incubated for the next 4 h. The viable cells containing NAD(P)H-dependent oxidoreductase enzymes reduced the MTT to blue formazan. The absorbance was measured spectrophotometrically at 590 nm. The measured absorbance was proportional to the number of the viable cells. The results are expressed as a % of non-treated control (TCP). For proliferation study, the cells were seeded at a density of 35 × 10^3^ cells on a 24-well plate and cultured for 24h at standard conditions. Then, the medium was discharged with a conditioned medium and three incubation times (3, 5 and 7 days) were performed. After each culture period, the amount of protein in each well was determined by Lowry’s protein assay estimation and the number of cells was determined using a calibration curve^47^. Moreover, the LDH release assay was used to determine cell death by assessment of LDH release from dead cells. The supernatants from the hemocompatibility test and culture medium from the cytocompatibility test were used for this study. The lactate dehydrogenase (LDH, fractional (S)-lactate:NAD+ oxidoreductase) was surveyed by direct measurement of NADH oxidation at 340 nm. LDH data were expressed as a % of the total LDH released from cells.

#### Antibacterial properties

The inhibition of bacterial growth was evaluated by measuring the turbidity of the cultured bacteria broth incubated with scaffolds. The cylinder-shaped specimens with a size of about 13 mm in diameter and 3 (± 0.02) mm thick were placed in Eppendorf tubes and then sterilized by UV-light exposure for 30 min. The tests were carried out according to the McFarland standards^48^ assuming the existence of a direct relation between the turbidity of cultured bacteria broth and the number of bacteria. Briefly, when the optical density of the bacterial suspension is 1.0 McFarland index (iMS), the number of bacteria is 3 × 10^8^ CFU mL^−1^. The experiments were performed using two different strains of bacteria: *Staphylococcus aureus* ATCC 29213and *Escherichia coli* ATCC 25922, and their initial concentration was set at 1.5 × 10^8^ CFU mL^−1^ (0.5 iMS). The bacteria were suspended in Trypticase Soy Broth (2.0 mL; Merck, Darmstadt, Germany) and incubated with scaffolds at 36 °C. After each hour, the optical density measurements were carried out using the DensiChEK Plus (BioMerieux, Montreal, QC, Canada). Bacteria incubated without specimens were used as a control. The maximum measuring range of this device was 4 iMS.

### In vivo validation and histological analysis

The in vivo experiment was carried out on a group of male New Zealand rabbits weighing 2.8–3.2 kg. The rabbits were purchased at the Experimental Medicine Center of the Medical University of Silesia in Katowice, fak. No.KCM/FPS/0041/06/20. Before the procedure, the animals' health was checked. The animals were under constant veterinary supervision and were given a vaccine: Castomix by Pharmagal Bio against Myxomatosis (MXT) and rabbit hemorrhagic disease (RHDV). All research protocols were approved by the Local Ethics Committee of the University of Life Sciences in Lublin No. 104/2017, and the experiment was conducted in accordance with the provisions on animal protection. The study is reported in accordance with ARRIVE guidelines. The animals were placed in the animal facility of the Experimental Medicine Center of the Medical University of Lublin. During this time, their natural habits were monitored and the temperature of each animal was measured daily. The general condition of the rabbits was very good, with no clinical signs of disease. The daily measured body temperature was within the reference range.

For 7 days after the herd was introduced to the Vivarium, their body temperature was measured, and the food intake and the behavior of the animals were observed. After a week's adaptation, the rabbits were prepared for surgery. After weighing, each individual was premeditated. On the day of surgery, each animal in the group was sedated by intramuscular injection (Domitor-Orion Corporation, Fin-land) of medetomidine (0.5 mg/kg) and Butomidor (Richter Pharma, Austria) butorphanol (0.2 mg/kg), depending on their weight. Then, after about 15 min, a mask was put on in order to administer inhalation anesthesia (isofluorane).

The period of anesthesia for each individual lasted about 30 min. After the rabbit was immobilized, the skin was shaved and disinfected with alcohol and iodine. The material for implantation was prepared according to the recommendations.

The skin incision was made parallel, in the intercostal area, in the middle of the latissimus dorsi muscle length, 3 cm above the dorsal line. Subcutaneous tissue and fascia were dissected in the same line and the prepared material was placed (cylindrical shape height 1 cm, diameter 1.5 cm). Two materials were implanted into one organism (one on the left and one on the right side). The implantation site was closed with a mattress suture using Dexon 3-0.

After the operation, all the rabbits could move freely in the cages without additional dressings in the area operated on. In order to minimize the risk of infection and reduce postoperative discomfort, an antibiotic and an anti-inflammatory drug (gentamicin 5 mg/kg and meloxicam 0.4 mg/kg) were administered for 5 days after the procedure.

In the postoperative period, mild swelling was observed around the skin suture in most rabbits. After two weeks, all the operated animals were in good general condition. Three months after surgery, all the rabbits were sacrificed. First, animals were anesthetized intramuscularly and sedated by intramuscular injection of medetomidine (0.5 mg/kg) and butorphanol (0.2 mg/kg) depending on their weight.

The rabbits were then sacrificed by barbiturate injection. Tissue fragments with a margin (3 cm × 3 cm × 3 cm) were taken from the implantation site along with the implanted material and placed in a buffered paraformaldehyde solution at pH 7.4. All the samples were placed in appropriate transporters and accurately described according to the implanted material.

The tissue samples were immediately fixed in 10% buffered formalin, processed routinely for histopathology using paraffin method, cut at 5 µm, and stained with Mayer’s haematoxylin and eosin. The samples were evaluated blindly by an experienced pathologist (IOD). Microphotographs were prepared using an Olympus BX43 microscope (Tokyo, Japan), equipped with Olympus SC 180 camera (Hamburg, Germany) and cellSens software (Olympus).

### Statistical analysis

Statistical analysis of the data was performed using commercial software (SigmaPlot 14.0, Systat Software, San Jose, CA, USA). The Shapiro–Wilk test was used to assess the normal distribution of the data. All the results were calculated as means ± standard deviations (SD) and statistically analyzed using one-way analysis of variance (one-way ANOVA). Multiple comparisons versus the control group between means were performed using the Bonferroni t-test with the statistical significance set at p < 0.05.

## Conclusions

Collagen is a protein with excellent biocompatibility. However, it can be resorbed in the body very easily. Phenolic acids, such as caffeic, ferulic and gallic acid, were studied as potential collagen modifiers. The biological properties of scaffolds based on collagen modified by phenolic acids were studied. The results showed that the addition of phenolic acids provides antioxidant activity (RSA in the range of 85–91%). Moreover, all of the scaffolds were non-hemolytic and safe to be used in contact with blood. Based on the results of the histopathological examination, it can be concluded that collagen is modified with caffeic acid and gallic acid faster and easier resorbs than when modified with ferulic acid implantation and does not generate an inflammatory process. Based on the results, it may be assumed that collagen scaffolds based on caffeic acid and gallic acid are the most suitable for biomedical application purposes.

## Data Availability

The datasets used and/or analysed during the current study available from the corresponding author on reasonable request.

## Abbreviations

ABS: Absorbance
ANOVA: Analysis of variance
CA: Caffeic acid
Coll: Collagen
DPPH: 2,2-Diphenyl-1-picrylhydrazyl reagent
FA: Ferulic acid
GA: Gallic acid
HE: Mayer’s haematoxylin and eosin
hFOB: Human fetal osteoblast cell
iMS: McFarland index
LDH: Lactate dehydrogenase
MIB: Minimum bactericidal concentrations
MIC: Minimum inhibitory concentrations
MTT: 3-(4,5-Dimethylthiazol-2-yl)-2,5-diphenyltetrazolium bromide assay
MXT: Myxomatosis
RBC: Red blood cells
RHDV: Rabbit hemorrhagic disease vaccine
ROS: Reactive oxygen species
RSA: Radical scavenging activity
SEM: Scanning electron microscope
SD: Standard deviations
TCP: Tissue culture plate
